# Transcriptomic and physiological effects of superabsorbent polymer seed coating on maize under drought stress

**DOI:** 10.3389/fpls.2026.1736004

**Published:** 2026-02-05

**Authors:** Akram Abdolmaleki, Hendrik Bertram, Peter Dapprich, Elena Meininghaus, Susann Michanski, Michaela Schmitz, Armin O. Schmitt, Mehmet Gültas

**Affiliations:** 1 Faculty of Agriculture, South Westphalia University of Applied Sciences, Soest, Germany; 2 Breeding Informatics Group, Georg-August University, Göttingen, Germany; 3 Center for Integrated Breeding Research (CiBreed), Georg-August University, Göttingen, Germany

**Keywords:** abiotic stress, drought-regulated genes, germination, mRNA-seq analysis, SAP, seed coating, *Zea mays*

## Abstract

Drought stress severely impairs maize germination and early seedling growth, posing a significant threat to global food security. To address this, superabsorbent polymers (SAPs) are being explored as an effective seed-coating method to improve water availability during the crucial germination phase. However, their comparative efficacy and underlying molecular mechanisms remain insufficiently understood. In this study, we evaluated the effects of three distinct SAPs, two fossil-based (MERCK, SWT) and one natural-based (ABG), on maize germination and seedling development under controlled drought conditions. We integrated physiological (germination rate and NA^+^), biochemical (total phenol content), and transcriptomic (mRNA-seq) analyses to provide a comprehensive multi-level assessment. Physiologically, among all SAPs, the MERCK was the most effective, resulting in the highest proportion of normal seedlings and the fewest abnormal seedlings. In contrast, the SWT treatment was detrimental, increasing the proportion of abnormal seedlings, suggesting phytotoxic effects. Biochemically, all SAP treatments resulted in elevated seedling sodium (Na^+^) content, indicating potential secondary ionic stress. Transcriptomic analysis further elucidated these observations, revealing a set of differentially expressed genes, including those involved in stress response (*BADH*, *FACT*, *XCP2*), SAP-specific response (*DRB5*, *RAF35*, *EDR1*), and combined salt/drought stress (*WRKY47*, *DTX20*), as promising candidate biomarkers for stress assessment and breeding. Our research highlights the nuanced efficacy of SAPs; specifically, the MERCK SAP yielded more favorable outcomes, while other formulations occasionally caused unexpected phytotoxicity. The identified gene expression patterns not only mechanistically explain the observed physiological responses but also offer a valuable panel of molecular biomarkers. These markers can be used to screen novel SAP applications, such as seed coatings, and to breed stress-resilient maize cultivars.

## 1 Introduction

Maize (*Zea mays* L.) is one of the most important cereal crops globally, serving as a staple food and a critical source of feed and fuel (Ji et al., 2025; Xuhui et al., 2022). Its importance is growing along with the increasing global population and the rising demand for agricultural products (Rida et al., 2021). However, maize production is increasingly challenged by both biotic and abiotic stress factors (Jiang et al., 2023; Bheemanahalli et al., 2022). Among abiotic constraints, drought is a leading factor that drastically reduces global crop productivity, affecting maize more severely than other cereals due to its high sensitivity to water deficits and the potential to reduce yields by over 50% (Jiang et al., 2023; Li et al., 2021; Rida et al., 2021).

Climate change has further intensified the frequency and severity of drought, salinity, and high-temperature stress events, compounding their adverse effects on seed germination, seedling establishment, and overall agricultural productivity (Li et al., 2021). Among all developmental stages, seed germination is particularly vulnerable to drought, making it a focal point for understanding and enhancing early-stage stress resilience. Germination represents a critical transition in the plant life cycle, and any disruption during this phase can severely impair subsequent growth and reproductive success (Chen et al., 2025). In maize, the germination process sets the foundation for final yield outcomes and is strongly influenced by water availability. Changes in seed moisture content affect the structure and activity of macromolecules such as starch and proteins, which in turn regulate enzymatic functions essential for metabolic activation (Song et al., 2022). Drought stress during germination is especially detrimental, with its impact depending on both the severity of the stress and the plant’s genetic makeup (Manavalagan et al., 2023). Therefore, protecting this stage against drought stress is crucial for securing optimal crop establishment and productivity. The increased drought intensity driven by climate change underscores the urgent need for adaptive crop management strategies to enhance resilience (Ibrahimi et al., 2024).

Given the increasing unpredictability of rainfall and the expansion of cultivation, the adoption of adaptive agronomic interventions has become essential. Among these innovations, superabsorbent polymers (SAPs) have emerged as a promising solution to enhance drought tolerance, particularly during germination and early growth stages (Ibrahimi et al., 2024). These hydrophilic polymers, characterized by functional groups such as hydroxyl and carbonyl, are capable of absorbing and retaining water hundreds to thousands of times their own weight, thus functioning as localized micro-reservoirs in the root zone (rhizosphere) (Feng et al., 2020; Yang et al., 2022; Gunday et al., 2021; Ai et al., 2021). Both fossil-based and natural-based SAPs have been developed, offering varying physicochemical properties and performance in enhancing soil moisture and mitigating drought (Li et al., 2019). Numerous studies have demonstrated the effectiveness of SAPs in various cropping systems under water-deficient conditions (El Idrissi et al., 2023; Meleha, 2022; Li et al., 2019; Ruqin et al., 2015; Salvanati et al., 2018). For instance, their application in green beans under drought stress improved plant water status, chlorophyll content, and yield, while reducing irrigation needs by up to 50% (Feng et al., 2020) and similarly, integrating SAPs with reduced fertilizer input in maize cultivation maintained yield levels and curtailed nutrient leaching by as much as 64%, underscoring their utility in sustainable agriculture (Egrinya Eneji et al., 2013). In barley, SAPs not only restored physiological traits under drought stress but also reshaped the rhizosphere microbiome, partially reverting it to conditions seen under normal watering (Amina et al., 2021). Furthermore, the natural-based SAP variant demonstrated superior performance in spring maize cultivation by enhancing soil moisture retention, microbial activity, and crop yield, while serving as an eco-friendly alternative to plastic-based polymers that contribute to microplastic pollution (Pan et al., 2025). SAPs have also shown considerable potential in seed coating applications, where hydrogel-based formulations, such as those containing gellan gum combined with growth-promoting agents, have improved wheat seedling drought tolerance and survival rates (Tursynova et al., 2025). Additionally, SAP coatings have been effective in preserving seed viability under drought conditions without compromising fungicide efficacy (Gubisova et al., 2024). In leguminous crops like black gram, biopolymer coatings have been shown to significantly enhance drought resistance and seed quality, suggesting their potential as a pre-sowing treatment for arid environments (Vijayalakshmi et al., 2023). Overall, SAPs represent a flexible, environmentally compatible tool for improving water-use efficiency and crop productivity in the face of water scarcity (Hotta et al., 2014). Moreover, our recent findings revealed the nuanced nature of soil-applied SAPs: while they exerted a moderate effect on crucial plant traits, their efficacy was highly variable, with distinct differences among species and even between cultivars (Abdolmaleki et al., 2025). This variability, coupled with the unique localized action of seed-coated SAPs, underscores the need to investigate the precise molecular processes induced by this pre-sowing treatment.

Understanding these unique, localized molecular mechanisms in maize requires advanced tools, such as transcriptomic analysis via messenger RNA sequencing (mRNA-seq). Hence, in maize, RNA-seq has been widely used to investigate gene expression profiles in response to various abiotic stresses, including drought (Cho et al., 2022; Gao et al., 2024). Studies have demonstrated its utility across multiple tissues, including roots, shoots, and reproductive organs such as developing ears and tassels, revealing distinct gene expression patterns under water deficit conditions (Kakumanu et al., 2012). Notably, RNA-seq has enabled the identification of differentially expressed genes (DEGs) that increase in number as drought stress intensifies, shedding light on the complexity of stress-induced gene regulation (Zou et al., 2024). This ability to map regulatory networks provides a systems-level understanding of how maize perceives and responds to environmental challenges. Additionally, the decreasing cost of NGS has made RNA-seq more accessible for large-scale studies, enabling researchers to investigate the molecular basis of adaptation to and tolerance of drought in greater depth (Liu et al., 2021). These insights are crucial for designing breeding strategies that aim to develop maize cultivars with enhanced resilience to climatic stressors. Addressing the growing challenge of drought stress in maize cultivation, our study introduces a groundbreaking, multi-dimensional investigation into the role of SAPs in enhancing drought resilience. Unlike previous research, we conducted an in-depth comparison of three diverse SAPs, two fossil-based (MERCK and SWT) and one natural-based (ABG), specifically focusing on their impact during the crucial germination and early seedling establishment phases under drought stress. This comparative approach, particularly as seed coatings, offers a unique perspective on the efficacy of SAPs not explored in studies limited to single SAP types and restricted traits. To decipher the underlying molecular mechanisms, we employed transcriptomic profiling via mRNA-seq in drought-stressed seedlings, providing a powerful lens into the genetic pathways activated by SAP treatments. Subsequent Gene Ontology (GO) and Kyoto Encyclopedia of Genes and Genomes (KEGG) enrichment analyses further illuminated critical biological processes and metabolic pathways implicated in SAP-mediated drought responses. Our research pursued two key objectives: (i) to demonstrate the efficacy of SAPs as seed coatings in alleviating early seedling drought stress, and (ii) to identify promising drought-responsive genes for advanced molecular breeding. Through this innovative integration of physiological, transcriptomic, and functional analyses, our findings offer a profound and comprehensive understanding of SAP mediated drought resilience in maize, holding significant implications for sustainable agriculture in a changing climate.

## 2 Materials and methods

### 2.1 Plant material, SAP usage and experiment design

The maize cultivar (*Zea mays* L. *subsp. indentata (Sturtev.) Zhuk.*, accession number ZEA 3639, spring type, was provided by the Leibniz Institute of Plant Genetics and Crop Plant Research (IPK, Gatersleben, Germany). To investigate the effects of SAPs on seed germination and gene expression, an experiment was conducted with five treatment groups, as outlined in **Table 1**.

**Table 1:** Experimental treatment groups.

Treatment	Abbreviation	Description	Application rate (g/kg seeds)
Isonem Soil Water Trap	SWT	Fossil-based SAP (lot number: lot: 55874, Isonem, Turkey)	60
MERCK SAP	MERCK	Fossil-based SAP (product no: MKCR9032, MERCK, Germany)	30
AgroBioGel	ABG	Natural-based SAP (AgroBiogel GmbH, Austria)	6,900
Well-watered control	CN	No SAP, not subjected to drought stress	N/A
Drought-stress control	CS	No SAP, subjected to drought stress	N/A

This table presents the experimental setup, identifying the five treatment groups, including two fossil-based SAPs, one natural-based SAP, and two control conditions. It specifies product details and the application methodology for each SAP.

Three types of SAPs were evaluated in this study. Merck and SWT, which are synthetic fossil-based polymers composed of cross-linked sodium polyacrylate, and ABG, a bio-based superabsorbent derived from wood (lignin-based). The sodium polyacrylate structure of the synthetic SAPs contains sodium (Na^+^) as the neutralizing counter-ion, which can be exchanged or released upon hydration. Although the manufacturer did not disclose the specific ionic composition of the bio-based ABG, we evaluated its effects on seedling evolution and ion uptake, alongside those of the synthetic variants. For each SAP treatment, 30 coated seeds and 30 non-coated seeds (controls) were placed on individual germination paper sheets (Keimtestpapier gelb; 120 mm × 300 mm, 160 g/m^2^; Sartorius, Göttingen, Germany). SAP application rates followed manufacturer recommendations. Their amounts were normalized based on the distinct water absorption capacity of each polymer (See **Supplementary Table S1**) and the thousand-seed weight of the maize variety (TSW = 363 g) to ensure a comparable amount of available water per seed across the SAP treatments.

Each germination sheet for the SAP and CS treatments was moistened with 5 mL of distilled water to induce drought stress, while the CN sheets received 12 mL to represent well-watered conditions. The applied water volumes were determined based on the water-holding capacity of the germination paper, following standard water-soaking procedures (Rajendra Prasad, 2023). For the stress treatments (CS and SAPs), the appropriate moisture level was established through preliminary water-retention tests. All samples were incubated for 7 days in a climate-controlled chamber (BINDER KBW 720; Tuttlingen, Germany) at 25 *±* 1°C under a 16/8 h light/dark photoperiod, following standard protocols for maize germination tests (Rajendra Prasad, 2023). At the conclusion of the incubation period, germination percentage and seedling biochemical parameters (total phenol content and sodium content) were recorded and prepared for subsequent gene expression analysis.

### 2.2 Germination performance

To evaluate the effects of different SAP treatments on seed germination behavior, we quantified and compared the proportions of normal, abnormal, and non-germinated seedlings. Seedlings were classified as normal if the radicle (embryonic root) and coleoptile (protective sheath) exhibited a straight primary root and showed no signs of necrosis in the cotyledons or root tissues. Seeds that displayed no visible germination, with no radicle and coleoptile, were classified as non-germinated. Abnormal seedlings were identified based on morphological anomalies, including looping or twisting of the primary root or cotyledons, absence of shoot or root structures, or the presence of necrotic tissue (Rajendra Prasad, 2023) (See **Supplementary Figure S2**).

A contingency table was constructed for the germination categories (abnormal, non-germinated, and normal) across treatments, with each treatment comprising controls and SAPs applied to 30 seeds. Pairwise comparisons of germination categories (normal, abnormal, and non-germinated) across treatments were performed using the G-test (log-likelihood ratio test), implemented in the pairwiseNominalIndependence function from the rcompanion package (Mangiafico, 2016) in R (R Core Team, 2021). P-values for the pairwise comparisons were adjusted for multiple comparisons using the false discovery rate (FDR) method (Benjamini and Hochberg, 1995). The significance of the pairwise comparisons was assessed using an adjusted p-value threshold of *α* = 0.05. Compact letter display codes were generated for each comparison using the cldList function from the rcompanion package to visualize significant differences between treatments within each germination category (Mangiafico, 2016).

Error bars represent the standard error for each proportion, computed using the binconf function in the Hmisc package in R.

### 2.3 Total phenol content determination

Total phenolic content (TPC) was extracted from 200 mg of fresh, normal seedlings, with six replications. Samples were flash-frozen in liquid nitrogen and homogenized into a fine powder using a mixer mill (Retsch MM200, Haan, Germany) at 25,000 rpm for 10 min. The powder was subjected to a sequential extraction with three 1 mL aliquots of methanol (CH_3_OH 95% (w/v)). After each methanol addition, the mixture was vortexed and centrifuged at 5,000 rpm for 5 min at 4°C (Rotina 380R, Hettich, Germany). The resulting supernatants were pooled for analysis. TPC was determined using the Folin-Ciocalteu colorimetric method (Anuar, 2014). A reaction mixture containing 0.5 mL of the methanolic extract, 0.5 mL of Folin-Ciocalteu reagent, and 0.5 mL of deionized water was briefly vortexed. After 30 seconds, 5 mL of sodium hydroxide (NaOH 1% (w/v)) was added. The solution was vortexed again and incubated for 30 min at room temperature (21 *±* 1°C). Absorbance was measured at 760 nm using a UV-Visible spectrophotometer (UV-1800, Shimadzu, Germany). The absorbance values were used to quantify the total phenolic content by referencing a standard calibration curve prepared with gallic acid (*R*^2^ > 0.99) (**Supplementary Figure S1**).

### 2.4 Sodium content determination

For sodium (Na^+^) analysis, the procedure was performed according to the manufacturer’s instructions for the Horiba LAQUAtwin Na^+^ ion-selective meter (Hildesheim, Germany). Specifically, 200 mg of fresh tissue from normal seedlings (six replications) was flash-frozen in liquid nitrogen and homogenized (Rotina 380R, Hettich, Kirchlengern, Germany) at 25,000 rpm for 10 min to obtain a fine powder. The homogenate was resuspended in 0.5 mL of distilled water and incubated at room temperature for 30 min with continuous agitation to ensure complete ion extraction. Insoluble material was sedimented by centrifugation at 10,000 rpm for 15 min (Rotina 380R, Hettich, Germany), and the Na^+^ concentration in the supernatant was quantified using the ion-selective meter. Results were expressed as parts per million per gram fresh weight (ppm).

TPC data were analyzed using one-way ANOVA in R software, followed by Tukey’s *post hoc* test to identify pairwise differences. Residual normality was evaluated using the Shapiro–Wilk test (Shapiro and Wilk, 1965), while homogeneity of variance was assessed with a Bartlett’s test (Bartlett, 1937). For Na^+^ analysis, due to variance heterogeneity, Welch’s ANOVA (Welch, 1951) was applied, followed by Games-Howell *post hoc* tests (Lee and Lee, 2018) to determine significant differences between treatments. A significance level of *α* = 0.05 was used for all comparisons.

### 2.5 mRNA extraction, library preparation, and data analysis workflow

Total RNA was extracted from plant tissues using the TIANGEN RNAprep Pure Plant Kit (Cat. no. 4992237, Beijing, China). Briefly, plant samples were ground to a fine powder in liquid nitrogen. For each 100 mg of tissue, 450 *µ*L of buffer RL, containing 1% *β*-mercaptoethanol, was added. The mixture was then vortexed vigorously, and after an optional incubation at 56°C for 1–3 minutes, the lysate was transferred to an RNase-free filter column and centrifuged at 12,000 rpm for 2–5 minutes. The supernatant was collected for subsequent steps. To ensure high-quality RNA, a DNase I treatment was performed by adding 80 *µ*L of the DNase I working solution (10 *µ*L of DNase I stock and 70 *µ*L of nuffer RDD) to the RNA sample for 15 minutes at room temperature. The RNA was then purified by washing with buffer RW1 and buffer RW, followed by elution with 30-100 *µ*L of RNase-Free water.

Total RNA was extracted from plant tissues, and RNA integrity was assessed using the Bioanalyzer 2100 system (Agilent Technologies, Santa Clara, USA). Messenger RNA (mRNA) was purified from total RNA using poly-T oligo-attached magnetic beads. After RNA purification and fragmentation, first-strand cDNA was synthesized using random hexamer primers. The second strand cDNA was synthesized using dUTP instead of dTTP to maintain strand specificity. The directional library was then constructed by performing end repair, A-tailing, adapter ligation, size selection, amplification, and purification. The final library was quantified using Qubit and real-time PCR, and its size distribution was analyzed using the Bioanalyzer. Following library quality control, the libraries were pooled based on effective concentration and targeted data amount and subjected to Illumina sequencing. Sequencing was performed using the Sequencing by Synthesis method, where fluorescently labeled dNTPs, DNA polymerase, and adapter primers were added to the flow cell for amplification. As each sequencing cluster extended its complementary strand, the addition of each fluorescently labeled dNTP released a corresponding fluorescence signal. The sequencer captured these signals and, using computer software, converted them into sequencing peaks to obtain sequence information for the target fragment. The mRNA extraction and library preparation were performed by Novogene (Munich, Germany).

Initially, the raw sequence reads underwent quality control using fastp version 0.24.1 (Chen et al., 2018). Adapter sequences were automatically identified and removed via the –detect_adapter_for_pe parameter, and poly-G/poly-A tails were trimmed using –trim_poly_g and –trim_poly_x, respectively. Furthermore, reads were trimmed from the 3’-tails to the front using the options –cut_tail and –cut_mean_quality 20 to achieve window mean qualities above 20. Afterwards, reads shorter than 50 bases were discarded (–length_required 50). Finally, bases of reads were classified as unqualified if their phred-score was below 20 (–qualified_quality_phred 20), and reads were discarded if the number of unqualified bases exceeded 30% (–unqualified_percent_limit 30).

The reference genome Zm-B73-REFERENCE-NAM-5.0 and corresponding gene and protein annotations were downloaded from Ensembl plant (Yates et al., 2021; Jiao et al., 2017). Filtered reads were aligned to the genome using STAR version 2.7.11b (Dobin et al., 2012) with default parameters. Base and read quality statistics were generated using fastp and FastQC version 0.12.1 (Andrews et al., 2010). Alignment statistics were generated using the bamqc and rnaseq commands of Qualimap version 2.3 (Okonechnikov et al., 2015). Consequently, all statistics were gathered using MultiQC version 1.28 (Ewels et al., 2016) for evaluation. Following the quality assessment using MultiQC and Qualimap, all samples met the established quality thresholds, and consequently, no samples were excluded or flagged for further analysis.

Sorting of alignment files was done using Sambamba version 1.0.1 (Tarasov et al., 2015) and gene-level quantification of alignment files was determined using featureCounts version 2.0.8 (Liao et al., 2013). Gene counts were extracted, and genes were filtered out if they were not abundant, i.e., if they did not have a raw count of at least 10 in at least 70% of the samples. This filtering was performed to increase statistical power by reducing the number of tests, thereby enabling a less stringent multiple-testing adjustment. Differential expression analysis using DESeq2 version 1.46.0 (Huber and Michael, 2017) in R version 4.4.3 (R Core Team, 2021) was conducted with CS as the reference group, applying FDR adjustment for p-values (padj) and log_2_ fold change (LFC) shrinkage with apeglm (Zhu et al., 2018). Finally, for each comparison, we considered genes as differentially expressed if padj *<* 0.05 and if they were up- or downregulated by a factor of 2 or higher (*|*LFC*| >* 1). For each comparison, we performed functional enrichment for GO terms as well as KEGG Pathways on the DEG sets by using the gost function of the gprofiler2 R package (version 0.2.3) (Kolberg et al., 2020). Terms were considered significantly enriched if padj *<* 0.05. *Arabidopsis thaliana* orthologs and gene names were retrieved from MaizeGDB (MaizeGDB, 2025).

A heatmap visualizing gene expression, generated from z-scores calculated for each gene from the DESeq2 analysis (to account for differences in library sizes), was produced using the ComplexHeatmap package in R (Mangiola and Papenfuss, 2020). The z-score was computed for each sample and gene using the formula 1:

(1) $$Z_{s,g}=\frac{X_{s,g}-\mu_{g}}{\sigma_{g}}$$

where *X* represents the raw count value, *µ* is the mean expression value of the gene across all samples, and *σ* is the standard deviation of the gene’s expression across all samples.

## 3 Results

To comprehensively assess the impact of SAP treatments on seedling development, we analyzed a suite of physiological parameters (germination rate and Na^+^) alongside biochemical indices (TPC).

### 3.1 Germination performance

**Figure 1:**
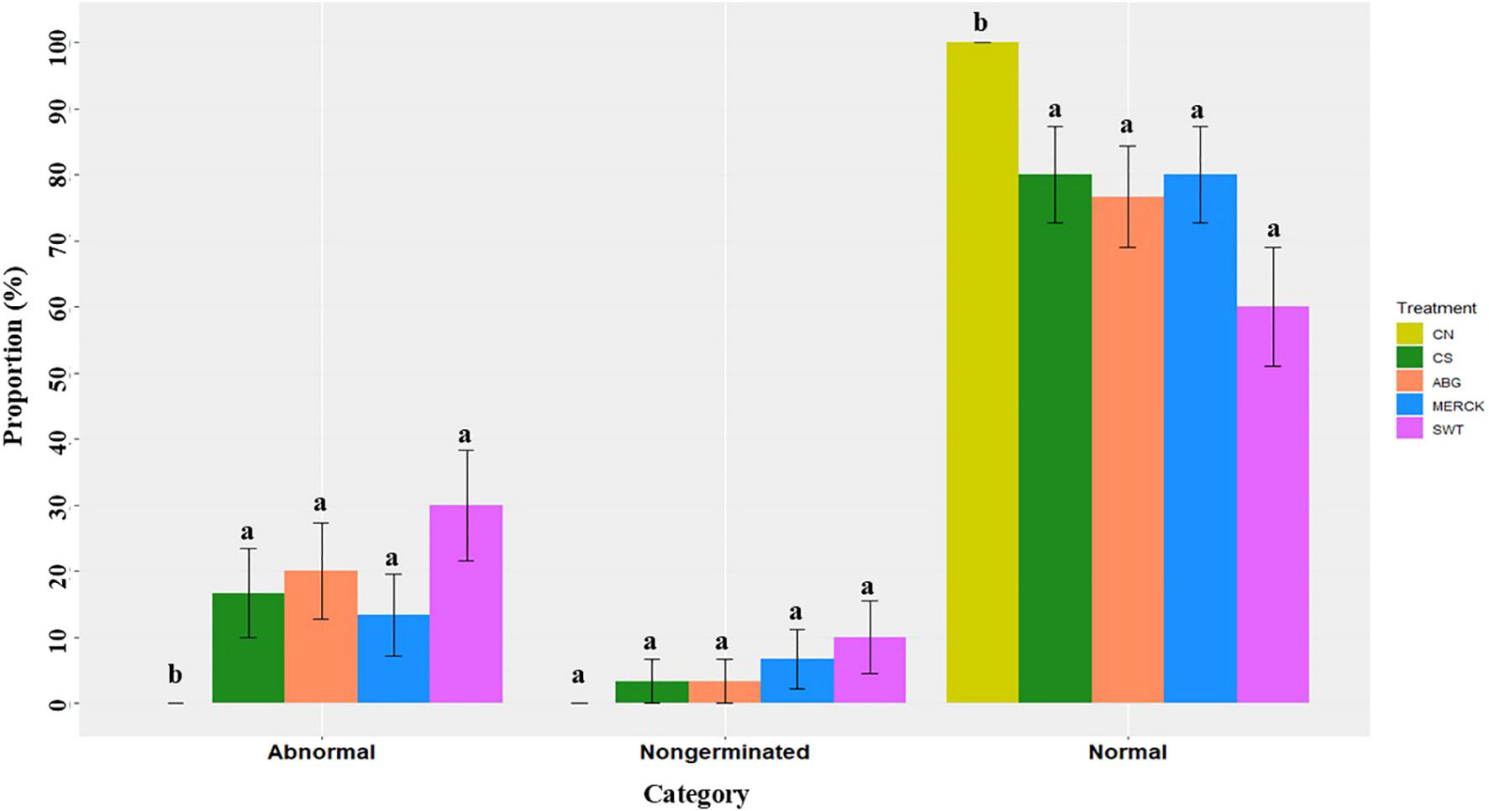
Effects of SAP treatments on maize seed germination. Bar plots show the mean proportions of normal, abnormal, and non-germinated seedlings across different treatments, with error bars representing the binomial standard error (SE) for each proportion. Each treatment included a sample size of 30 seeds. Distinct letters above the bars represent statistically significant differences (*α* = 0.05) between treatments within each germination category, based on pairwise comparisons using the G-test (log-likelihood ratio test) with FDR adjustment for multiple comparisons.

Germination success following SAP treatments was assessed by classifying seedlings into three categories: normal, abnormal, and non-germinated. The distribution of these outcomes is shown in **Figure 1**. An omnibus G-test was conducted to evaluate overall treatment effects on germination at a significance level *α* = 0.05. The results showed a reduction in the proportion of normal seedlings under all stress conditions, including SAP treatments and CS. Although there was no statistically significant difference among the SAP treatments, Merck showed a trend toward a higher germination rate (80%) than ABG (76%) and SWT (60%). In contrast, the SWT treatment negatively affected seedling development, resulting in a decrease in the proportion of normal seedlings and a corresponding increase in abnormal seedlings compared with the CN treatment leading to a 30 percentage points rise in non-germination. This elevated rate of morphological anomalies suggests a less favorable interaction under the tested conditions. Further details are provided in **Supplementary Table S2**. The ABG treatment performed at an intermediate level, with its germination profile not significantly different from that of CS. Regarding the non-germinated category, there were no statistically significant differences among any of the stress treatment groups. However, all stress treatments showed higher proportions of non-germinated seedlings than CN, with SWT showing the highest non-germination rate (10%) and significantly higher abnormal seedling rates. This suggests that while certain SAPs can support germination development, their ability to overcome initial germination inhibition under the applied stress conditions may vary fully.

### 3.2 Total phenolic content analysis

**Figure 2:**
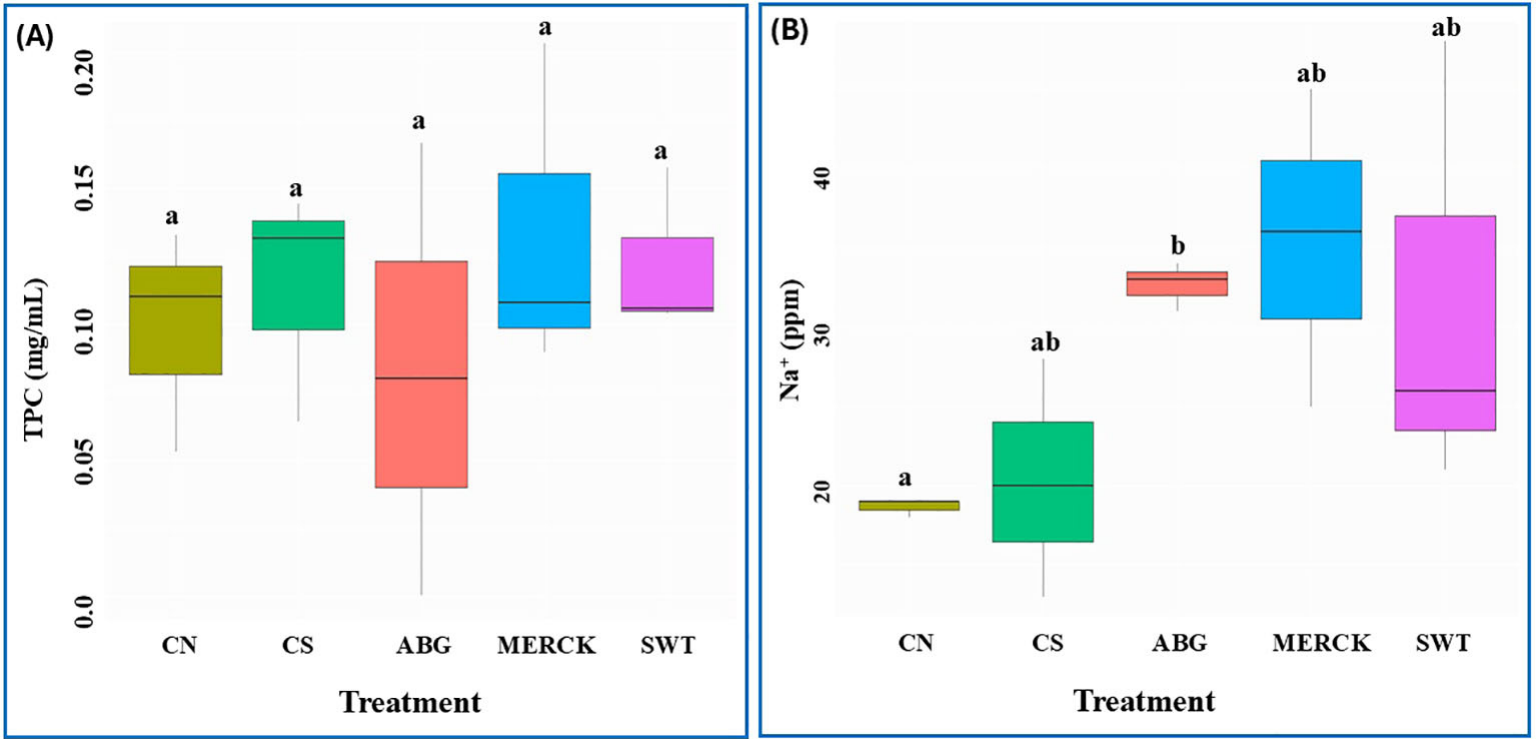
Total phenol and sodium content analysis. Box plots displaying the effects of treatments on **(A)** TPC and **(B)** NA^+^ content in seedlings for each treatment. For TPC, analysis was performed using One-way ANOVA followed by Tukey’s *post-hoc* test. For the NA^+^ content, due to variance heterogeneity, Welch’s ANOVA followed by the Games-Howell *post-hoc* test was applied. Distinct letters above the bars indicate statistically significant differences between treatments (*α* = 0.05).

To evaluate the biochemical impact of the SAP treatments, TPC, a common indicator of oxidative stress, was assessed in the seedlings. As illustrated in **Figure 2A**, statistical analysis revealed no significant differences in TPC levels among any of the treatment groups, including the controls. Despite this lack of statistical significance, some patterns were observed. Notably, the ABG treatment exhibited the lowest TPC values among all groups by 0.08 mg/mL, including the controls, potentially suggesting a stress-mitigating effect. The SWT treatment also resulted in TPC levels that were, on average, lower than the CS control. The MERCK treatment displayed an intermediate profile, with TPC levels generally positioned between those of the controls and the other SAPs. High variability in TPC levels was observed, particularly for ABG and MERCK. While SAPs appeared to modulate seedlings’ phenolic response, no statistically significant reduction in oxidative stress indicators was observed under the conditions of this study.

### 3.3 Na^+^ content

Sodium accumulation in seedlings was analyzed to evaluate the impact of SAP treatments onion uptake (**Figure 2B**). While all SAP treatments, on average, increased Na^+^ content in seedlings compared to controls, statistical analysis revealed the following specific patterns. The ABG treatment resulted in significantly higher Na^+^ levels compared to the CN control. In contrast, no significant difference was observed between the fossil-based SAPs (SWT and MERCK) and the controls, a finding that may be influenced by high variability within these treatment groups. However, the consistent numerical elevation of Na^+^ in SWT and MERCK, relative to the controls, suggests a tendency toward increased sodium uptake. This overall pattern of increased sodium accumulation, particularly the significant increase with ABG, could indicate an additional osmotic or salt stress induced by the SAPs, which may have contributed to the observed impacts on seedling development.

### 3.4 Transcriptome analysis

A total of 129 million to 177 million raw reads per sample were obtained across five treatments, with two biological replicates per SAP treatment and per control group. After adapter removal and filtering of low-quality sequences, over 97% of the resulting reads were classified as high-quality. When mapped to the *Zea mays* reference genome, between 92.16% and 95.04% of the high-quality sequences were successfully aligned, indicating efficient mapping to the reference genome (See **Supplementary Table S3**).

**Figure 3:**
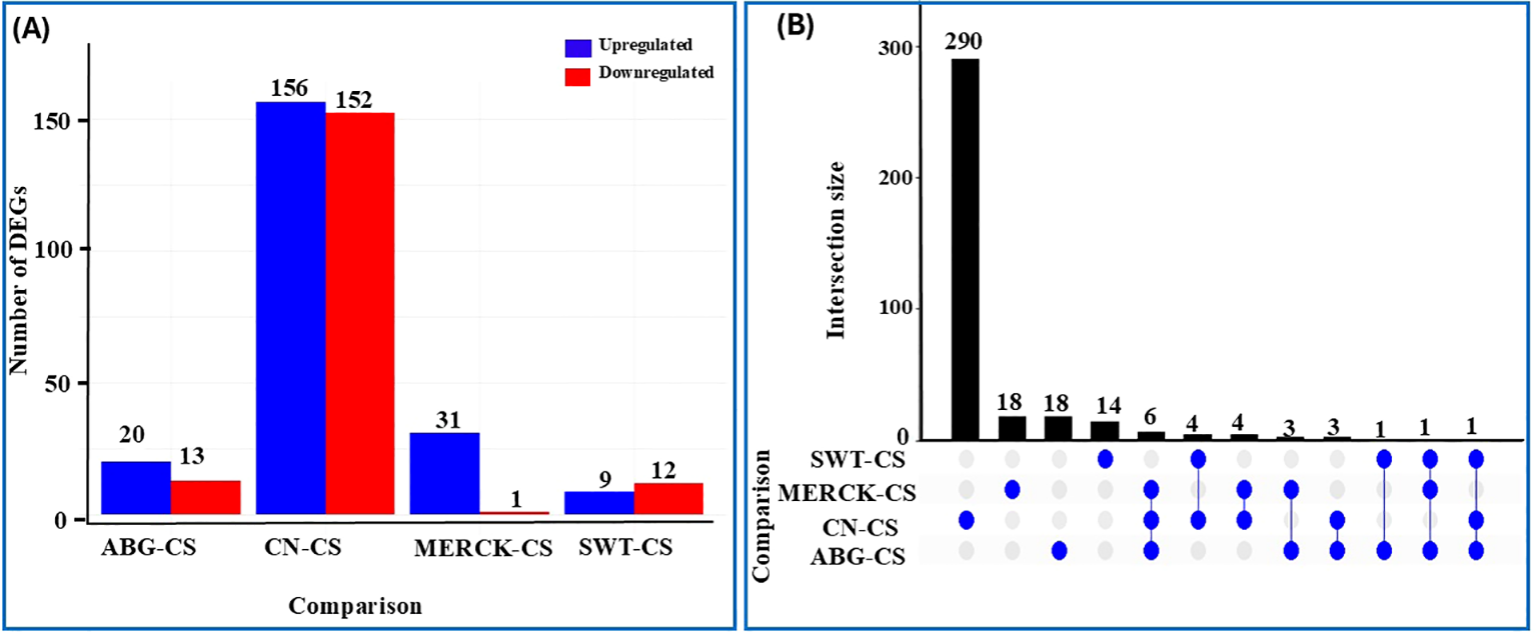
Transcriptomic variation across treatment groups relative to the CS reference group. Differential expression analysis was performed using DESeq2, where genes were considered significant at FDR-adjusted p-values (padj) *<* 0.05 and absolute log_2_ fold changes (|LFC|) *>* 1. **(A)** Number of DEGs identified in pairwise comparisons with CS, categorized by direction of change (Upregulated and Downregulated). **(B)** UpSet plot showing unique and shared DEGs among the pairwise comparisons with CS.

Transcriptome analysis revealed that the most profound transcriptional shift occurred in response to drought stress itself, with the CN vs. CS comparison yielding the highest number of DEGs with 156 upregulated and 152 downregulated (**Figure 3A**). The application of SAPs distinctly modulated this response. Specifically, while MERCK and ABG induced a similar total number of DEGs against the stress control (32 and 33, respectively), their expression patterns differed significantly; for instance, MERCK showed a higher proportion of upregulated DEGs (20 up, 12 down). In comparison, the ABG response was more balanced (20 up, 13 down). In stark contrast, the SWT vs. CS comparison yielded the lowest transcriptional response, with only 21 total DEGs, suggesting a suppressed or failed molecular adaptation (See **Supplementary Table S4**).

The distribution of common and unique DEGs, visualized in the UpSet plot (**Figure 3B**), further clarifies these distinct strategies. The CN vs. CS comparison identified 290 unique DEGs, underscoring the extensive and specific transcriptional reprogramming induced by drought. Among the SAPs, the most significant overlap involved six DEGs shared between the MERCK vs. CS and ABG vs. CS comparisons, suggesting some convergence in their molecular mechanisms. These two treatments also shared several DEGs with the primary drought response (CN vs. CS), indicating they modify existing stress pathways. In contrast, the minimal number of DEGs and limited overlap seen in the SWT treatment reinforce the conclusion of a muted and distinct response. The fact that only one DEG was shared across all three SAP treatments indicates that each polymer elicits a distinct molecular adaptation in the seedling.

Functional enrichment analysis provided further insight into these responses. GO enrichment analysis identified a limited number of significantly enriched terms exclusive to the MERCK vs. CS comparison, suggesting specific pathway modulation by this SAP (see **Supplementary Table S5**). Notably, no significant GO terms were identified for the broad-spectrum CN versus CS drought response, nor for the DEGs of the ABG and SWT treatments. Similarly, KEGG pathway analysis identified only four significantly enriched pathways, all exclusive to the SWT vs. CS comparison (**Supplementary Table S6**), with no significant pathways observed in any other contrast. This general lack of widespread functional enrichment, particularly for the overall drought response, suggests that the seedling’s molecular adaptations may involve subtle, diverse, or novel pathways not fully captured by current annotations, rather than broad, well-defined functional categories.

#### 3.4.1 Target genes analysis

**Figure 4:**
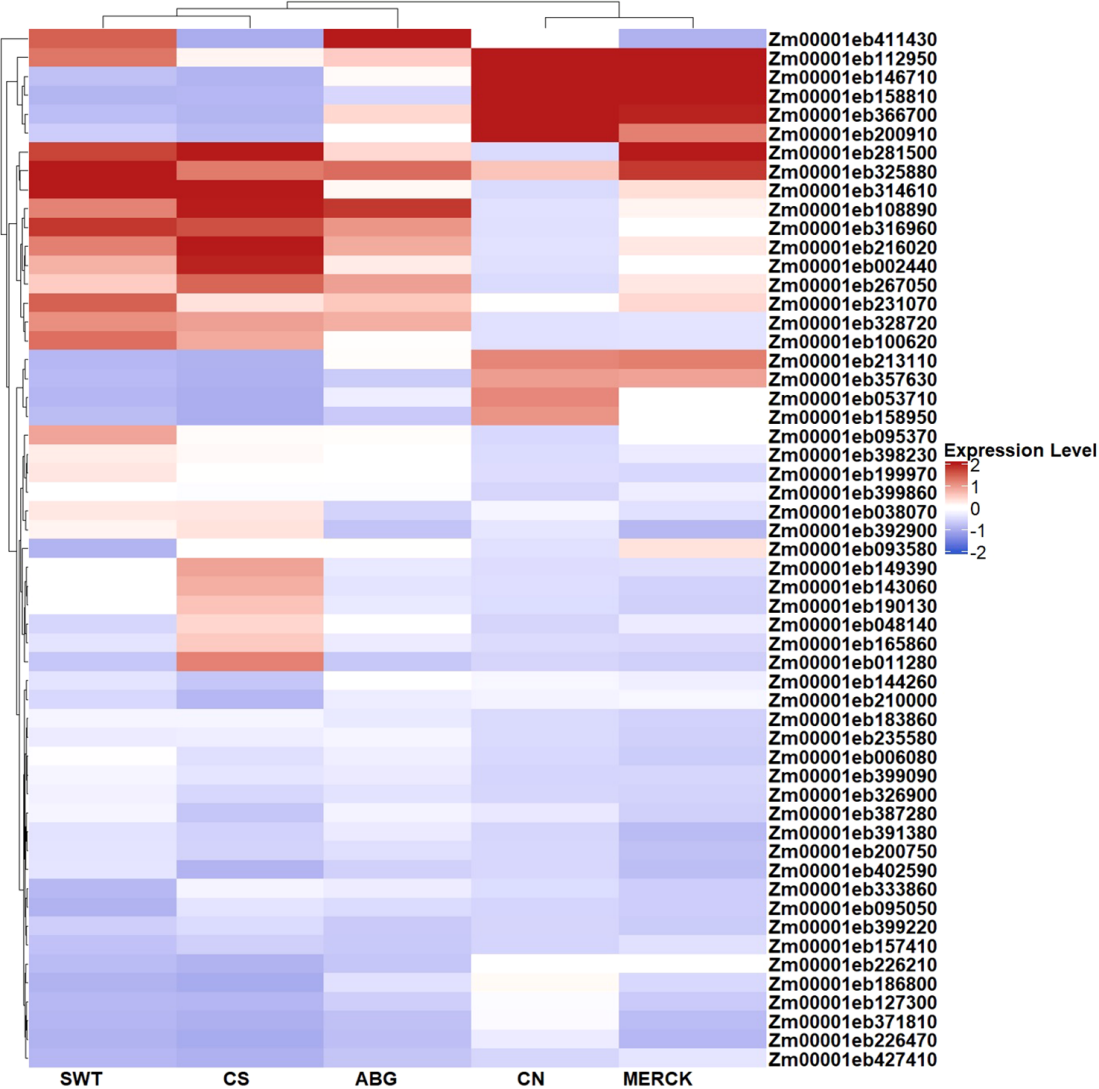
Heatmap of stress-responsive gene expression. Selected genes are annotated with GO terms related to salt stress, water deprivation, hydrogen peroxide catabolism, and antioxidant activity. Mean normalized expression values, averaged across biological replicates and standardized by row-wise z-scoring (calculated based on Equation 1), are shown. Hierarchical clustering of genes (rows) and treatments (columns) was performed using Euclidean distance and complete linkage (R package ComplexHeatmap). The symmetric color scale is centered at zero and reflects relative deviations from each gene’s mean expression across treatments.

Differential expression analysis across all treatment comparisons identified 363 unique DEGs (See **Supplementary Table S4**), of which 55 genes are directly involved in stress response pathways (See **Supplementary Table S7**). The heatmap in **Figure 4** illustrates expression profiles of representative DEGs showing the most pronounced regulation across treatments. Many of these genes are functionally annotated with roles in oxidative stress, osmotic adjustment, and hormonal signaling, highlighting their contribution to treatment-specific adaptation mechanisms.

Several clear expression patterns emerged such as *Zm00001eb411430*, a member of the *β-glucosidase* (*BGLU*) family associated with oxidative stress responses (Kannan et al., 2023), which was strongly induced under ABG (LFC = 7.06) but showed lower expression under SWT and minimal expression in MERCK and CN, indicating treatment-dependent modulation of oxidative stress defenses. Similarly, *Zm00001eb392900* (*EDR1*), a key regulator of drought signaling (Xie et al., 2023), displayed contrasting dynamics. It was downregulated in the MERCK vs.CS and ABG vs.CS groups but upregulated in the SWT and CN groups, indicating selective regulation depending on the treatment (**Table 2**).

**Table 2:** Differential expression of stress-responsive genes under drought.

Gene ID	Arabidopsis ortolog	Gene name	Log2FoldChange			
			CN vs. CS	MERCK vs. CS	ABG vs. CS	SWT vs. CS
Zm00001eb411430	AT5G25980	BGLU37/TGG2	0.06	0.00	7.06^*^	0.00
Zm00001eb392900	AT1G08720	EDR1	-0.11	0.00	-4.21^*^	0.00
Zm00001eb267050	AT1G20850	XCP2	-2.32^*^	-0.01	0.00	0.00
Zm00001eb053710	AT5G65550	LPE1	7.36^*^	0.00	0.00	0.00
Zm00001eb038070		EREB172	0.20	0.00	-2.01^*^	0.00
Zm00001eb011280	AT2G02850	PCY	-5.04^*^	0.00	0.00	0.00
Zm00001eb235080	AT5G03200	LOG2	12.58^*^	0.00	0.00	12.61 ^*^
Zm00001eb146710	AT5G63560	FACT	6.23^*^	0.00	0.00	0.00
Zm00001eb124150	AT5G57840	GGES	6.28^*^	0.00	0.00	0.00
Zm00001eb096360			-6.60^*^	0.00	0.00	0.00
Zm00001eb304050	AT3G46720		-8.58^*^	0.00	0.00	0.00
Zm00001eb019640	AT3G13840	GRAS family transcription factor	0.49	0.00	-3.42^*^	0.00
Zm00001eb210970	AT5G09950	MEF7	0.062	0.00	-5.77^*^	0.00
Zm00001eb005700	AT5G26200		0.33	0.88	1.52^*^	1.41 ^*^
Zm00001eb102530	AT3G26932	DRB3	0.19	1.21^*^	1.36^*^	0.00
Zm00001eb092510	AT1G54790	GGL16	0.00	10.10^*^	0.00	0.00
Zm00001eb316690	AT3G56150	EIF3C	-0.02	0.00	0.00	-5.98^*^
Zm00001eb403320	AT1G75330	OTC	-0.13	0.00	0.00	-6.83^*^
Zm00001eb074830	AT4G17460	HAT2/HAT1	1.44	2.42^*^	0.00	0.00
Zm00001eb214150	AT5G61840	GUT1	-0.03	6.58^*^	0.00	0.00
Zm00001eb260760	AT2G17650	AAE2	0.15	3.80^*^	0.00	0.00
Zm00001eb129690	AT4G01720	WRKY47	-0.05	0.00	0.00	-8.97 ^*^
Zm00001eb093580	AT1G33100	DTX20	-0.05	0.00	0.00	-5.13^*^
Zm00001eb333860	AT5G01070		-0.13	0.00	0.00	-3.16^*^
Zm00001eb095050	AT3G47160		-0.14	0.00	0.00	-8.34 ^*^
Zm00001eb337870	AT5G66760	SDH1-1	-1.65	0.00	3.43^*^	0.00
Zm00001eb360940	AT2G32510	MAPKKK17	0.10	0.00	-2.32^*^	0.00
Zm00001eb147730	AT5G57610	RAF35	0.08	11.03^*^	9.62^*^	0.00
Zm00001eb102530	AT5G41070	DRB5	0.19	1.21^*^	1.36^*^	0.00
Zm00001eb235080	AT5G03200	LUL1	12.58^*^	0.00	0.00	12.61 ^*^
Zm00001eb043940	AT4G00180	YAB3	0.07	0.00	11.12^*^	0.00
Zm00001eb264810			0.05	4.55^*^	4.49^*^	3.82^*^

*Arabidopsis thaliana* orthologs and their gene names are listed for functional context where available. Negative log2 fold changes (LFC) denote downregulation (blue) and positive LFC-values denote upregulation (red) relative to CS. Asterisks (*) indicate significant differences in LFC, based on padj *<* 0.05 and *|*LFC*| >* 1.

Genes linked to cellular stress signaling also showed consistent patterns. *Zm00001eb267050* (*XCP2*) was upregulated in CS and across all SAP treatments but remained low in CN, highlighting their stress-induced activation. Transport-related processes were also represented. *Zm00001eb053710* (*AT5G25420*, *LPE1*), which encodes a nucleobase–ascorbate transporter (Liao et al., 2025), was significantly upregulated in CN relative to CS (LFC = 7.36), indicating a potential role in ascorbate mobilization during early stress recovery rather than under active drought conditions.

Several transcriptional regulators were differentially expressed. *Zm00001eb038070*, encoding an *EREB172* (*AP2/EREBP* transcription factor 172) from *AP2/ERF*-domain transcription factor family, was strongly downregulated under ABG, consistent with the established roles of *AP2/ERF* in ABA- and ethylene-mediated abiotic stress signaling (Su et al., 2025). In contrast, *Zm00001eb011280* (*AT2G02850*, *plantacyanin*), a component of the *PIF1–miR408–PCY* axis that promotes a low-GA/high-ABA state and represses early germination (Jiang et al., 2021), as well as a regulator of ROS accumulation in guard cells (Yang et al., 2024), was significantly downregulated in CN. The observed repression, consistent with the 100% germination rate, suggests that reduced *PCY* levels under favorable conditions enable a mechanism that supports growth.

Within the broader DEG set, several genes emerged as consistently upregulated in CN. These included *Zm00001eb235080* (*AT5G03200*, *LOG2*), a cytokinin activator essential for meristem maintenance and hormonal balance (Kuroha et al., 2009) (LFC = 12.58, **Supplementary Table S4**). *Zm00001eb146710* (*AT5G63560*, *FACT*), which catalyzes the transfer of caffeoyl and feruloyl groups to primary alcohols, thereby cross-linking aliphatic and phenolic domains during suberin assembly (Wei et al., 2020), and *Zm00001eb124150*, LFC = 6.28 (**Supplementary Table S4**), (*AT5G57840*, *GGES*), a *BAHD*-family acyltransferase that esterifies phenylacetic acid, a phenylalanine-derived phenolic compound, onto glucuronosylglycerol, thus linking phenolic metabolism with lipid-derived pathways (Simpson et al., 2024).

In contrast, some genes were consistently downregulated, including *Zm00001eb096360*, a member of the papain-like cysteine protease (*PLCP*) family, the most abundant group of cysteine proteases in plants with key roles in biotic and abiotic stress responses, growth, and senescence (Zhai et al., 2021), which here showed lower expression under SAP treatments. Also downregulated was *Zm00001eb304050* (*AT3G46720*), a glycosyltransferase gene which is known to play critical roles in plant defense and stress tolerance, as aglycones often include plant hormones, defense-related secondary metabolites, and xenobiotics such as herbicides (Yu et al., 2017). The observed downregulation in CN vs. CS (equivalently, the upregulation in CS vs. CN) indicates activation of defense mechanisms under drought stress in maize.

The DEG profiles revealed that the SAPs elicited both a core stress response and highly polymer-specific adaptive programs (**Figure 3B**; **Supplementary Tables S4-S7**). The most striking evidence for a conserved mechanism is the single gene, *Zm00001eb264810*, that was commonly upregulated across all three SAP treatments, with LFC values of 4.49 for ABG, 4.55 for Merck, and 3.82 for SWT (**Table 2**). This gene encodes a chloroplast-localized enzyme involved in thiamine biosynthesis (MaizeGDB, 2025). Its consistent induction suggests that increasing thiamine supply, a crucial cofactor for energy metabolism and ROS detoxification, is a universal and fundamental strategy for buffering the stress imposed by these polymers. Beyond this universal response, partial overlaps in the DEG sets highlight more nuanced similarities. For example, *Zm00001eb102530* was shared between MERCK and ABG, while *Zm00001eb005700* was common to ABG and SWT, indicating that different SAPs can recruit conserved modules for metabolic regulation or stress signaling. Ultimately, the unique DEGs identified in each SAP treatment represent candidate genes that likely reflect the direct pathways and mechanisms employed by those SAPs to shape the observed physiological responses. For instance, the dysregulation of specific developmental regulators, such as *Zm00001eb019640* and *Zm00001eb210970*, in the ABG and SWT treatments aligns directly with their high rates of seedling abnormality. Similarly, the specific induction of the antioxidant-related gene *Zm00001eb411430* in ABG provides a direct mechanistic link to its distinct TPC profile. These findings demonstrate that while all SAPs trigger a foundational stress response, the success or failure of a given polymer is determined by a unique secondary layer of gene regulation that ultimately shapes the germination and stress tolerance phenotype.

## 4 Discussion

As a staple crop crucial for global food security, maize production is severely threatened by drought, one of the most detrimental abiotic stresses limiting its cultivation. The susceptibility of maize to water deficits negatively affects its entire life cycle, from vegetative growth and biomass production to reproductive organ formation and final yield parameters (Badr et al., 2020; Ochoa-Chaparro et al., 2025). The germination and seedling stages are particularly vulnerable, as maize is more sensitive to drought than other cereals during this early phase of development (Rida et al., 2021). At this critical stage, drought stress has been shown to reduce the germination rate, potential, and index of maize seeds, while also inhibiting the growth of both the plumule and radicle, thereby compromising successful crop establishment (Rida et al., 2021). Therefore, addressing these early-stage vulnerabilities is crucial for enhancing maize resilience and productivity.

### 4.1 Germination performance, physiological and biochemical responses

Our results indicate that drought stress negatively affected maize germination quality, reflected in a higher proportion of abnormal and non-germinated seeds in CS compared to CN (**Figure 1**). This finding is consistent with the established mechanism in which drought reduces osmotic potential, preventing sufficient water uptake by the seed. This impairment slows the essential metabolic processes required for germination, thereby reducing germination success rates (Jaleel et al., 2009). Indeed, our observation of reduced germination success under stress aligns directly with previous studies on maize (Khodarahmpour, 2011).

In our study, the MERCK SAP showed a tendency to support normal seedling development, with a 20 percentage points higher germination rate than SWT and four percentage points higher than ABG, and fewer seedling abnormalities than other Stressed treatments (by 13%), suggesting a potentially beneficial role for MERCK under drought conditions. In contrast, the SWT treatment had a clear adverse impact, with the highest rate of abnormal seedlings (30%) and non-germination (10%), and the lowest proportion of normal seedlings (60%), suggesting that any potential benefits of water retention from SWT were negated by its inhibitory or incompatible properties, thereby exacerbating stress on germinating maize seeds, the ABG treatment was performed at an intermediate level of complexity. The general trend of slightly higher non-germinated seeds in SAP-treated groups compared to controls hints that initial germination inhibition under stress remains a challenge that these SAPs did not fully overcome.

Abiotic stresses, including drought, are known to induce oxidative stress in plants by overproducing ROS, which can damage vital cellular components. In response, plants typically enhance their antioxidant systems to tolerate this stress better, often leading to the accumulation of secondary metabolites, such as phenolic compounds (Rahimi et al., 2021). This defensive accumulation is a recognized marker of drought response, with numerous studies confirming that water deficit effectively increases TPC in plants (Mohammadi et al., 2020). Higher phenolic levels often correlate directly with enhanced antioxidant capacity (Mohammadi et al., 2020). However, contrary to the expected trend, our study did not find a significant difference in TPC levels across the treatments. This observation suggests that SAPs may have reduced the severity of drought stress, thereby limiting the triggers that typically stimulate phenolic production. However, these findings should be interpreted with caution. Since TPC was measured only at a single developmental stage (7-day seedlings), it is possible that the peak stress response occurred at a different time. Thus, while the stable TPC levels are consistent with a stress-mitigation hypothesis, they do not definitively prove that oxidative stress was entirely absent.

A critical finding of our study is that seedlings treated with SAPs exhibited significantly elevated internal sodium levels, with concentrations increasing by at least 77% compared to CN (18 ppm), reaching 32 ppm in ABG and SWT, and 35 ppm in Merck. This accumulation is consistent with the release of sodium ions from the polymer matrix during hydration, particularly for the sodium polyacrylate-based SAPs (Merck and SWT), which rely on Na^+^ dissociation for swelling. Interestingly, despite being a bio-based material, the ABG treatment also resulted in increased seedling Na^+^ levels. While this suggests that the bio-polymer formulation may contribute ions to the rhizosphere, we cannot rule out the possibility that SAPs altered the osmotic dynamics of the root zone, thereby influencing passive ion uptake mechanisms. Regardless of the specific mechanism, this inadvertent elevation of sodium imposed a secondary salinity stress on top of the primary drought condition. This dual-stress scenario is particularly detrimental, as high Na^+^ concentrations can disrupt osmotic and ionic homeostasis, inducing secondary oxidative stress (Bosnic et al., 2018). Maize is moderately sensitive to salinity, with germination and early seedling stages particularly vulnerable (Farooq et al., 2015; Khalid et al., 2023). This increased sensitivity can manifest not only as a failure to germinate but also as impaired post-germination development. For example, Hernandez et al. (2018) found that although germination percentage was unaffected by combined osmotic and metabolic stress, subsequent seedling growth and fresh weight were significantly reduced. These findings align with our observations of increased seedling abnormalities in SAP treatments. Thus, the elevated sodium load associated with SAP application, including the bio-based ABG, likely contributed significantly to these developmental setbacks, transforming what was intended as a drought-mitigation tool into an additional source of compound stress for maize seedlings.

### 4.2 Transcriptome analysis: divergent strategies for growth and stress response

Our transcriptome analysis provides a comprehensive understanding of how the distinct physiological outcomes observed in control (CN) and SAP-treated (MERCK, ABG, SWT) seedlings are driven by fundamentally different impacts on gene expression, affecting RNA metabolism, stress signaling, metabolic adjustments, and developmental regulation.

#### 4.2.1 CN treatment: drought stress response genes

Under well-watered control conditions, CN seedlings exhibit optimal growth (perfect germination, low Na^+^, low TPC), supported by gene expression profiles indicative of efficient resource allocation and robust cellular integrity.

The significant overexpression of *Zm00001eb146710*, likely a beneficial *BAHD acyltransferase* ortholog (*At5g63560* or *FACT*), suggests enhanced synthesis of protective suberin and associated alkyl caffeate waxes (Kosma et al., 2012; Nomberg et al., 2022). The upregulation in CN vs. CS corresponds to downregulation in CS vs. CN, and vice versa. Notably, reduced expression of this gene under stress in CS may limit protective suberin deposition, potentially compromising barrier functions that regulate water and ion flux as well as seed coat integrity (Choi et al., 2023). This could help explain the higher sodium accumulation, reduced germination rate, and greater frequency 4of abnormal and non-germinated seeds observed in CS.

Additionally, *Zm00001eb053710*, an ortholog of *Leaf Permease1* (*LPE1*) in the Nucleobase–Ascorbate Transporter (NAT) family (Argyrou et al., 2001; Guo et al., 2022), is highly expressed, consistent with increased transport of the nucleobase substrates xanthine and uric acid that support nucleotide recycling and stress physiology, including seed germination and drought tolerance (Liao et al., 2025). In CS, its downregulation could therefore contribute to impaired germination. Guo et al. (2022). demonstrated that the cotton *LPE1* ortholog is linked to salt tolerance; hence, lower expression in CS is also consistent with the elevated Na^+^ observed in this line.

Furthermore, *Zm00001eb267050*, an ortholog of *Xylem Cysteine Peptidase 2* (*XCP2*) involved in programmed cell death (PCD) during xylem differentiation and stress responses (Nakaba et al., 2015; Zhang et al., 2014; Avci et al., 2008), is significantly downregulated in CN but upregulated in CS relative to CN. Given *XCP2*’s role in tracheary, element autolysis, and higher expression in CS, it may intensify PCD under stress, perturbing early vascular maturation and hydraulic control. This aligns with the poorer seed performance of CS, lower germination, and more abnormal/non-germinated seedlings, and may also contribute to its elevated Na^+^ via less selective ion movement. By contrast, dampened *XCP2* expression in CN may limit stress-induced PCD and help preserve seedling integrity. This expression divergence in maize aligns with the *PRN–XCP2* regulatory module described in Arabidopsis, in which *PRN1* participates in blue-light and ABA responses that influence germination and early seedling development, and *PRN2* stabilizes *XCP2* by inhibiting its autolysis, as shown by PLCP-activity profiling and recombinant-protein assays (Zhang et al., 2014).

#### 4.2.2 MERCK treatment: coordinated adaptation

The higher performance of the Merck treatment compared to other SAPs, resulting in the highest proportion of normal seedlings, suggests a coordinated metabolic and regulatory strategy that appears to balance stress management with growth promotion. Several genes activated in MERCK were associated with RNA metabolism and the control of stress signaling. For instance, the *DRB5* (*Zm00001eb102530*), a double-stranded RNA-binding protein involved in miRNA-mediated gene silencing under stress (Eamens et al., 2012; Behera et al., 2025; Sawano et al., 2016), was significantly upregulated (LFC = 1.02), indicating activation of stress-associated RNA silencing pathways. Both MERCK and ABG treatments induced stress responses, as evidenced by upregulation of *RAF35* and *Zm00001eb147730*, LFC = 11.03 and 9.62, respectively, components of osmotic stress/ABA signaling (Lin et al., 2020). However, MERCK demonstrated a crucial difference in downstream regulation. The expression of *Zm00001eb392900* (*MAPKKK EDR1*), a negative regulator of stress responses (Tang et al., 2005; Christiansen et al., 2011), showed less severe repression than other treatments, suggesting a finely tuned balance that activates stress responses while preventing self-destructive responses.

Transcriptomic analysis revealed that metabolic and osmotic adjustment strategies were activated in MERCK. Specifically, *Zm00001eb214150* (homologous to *GUT1* and *GUT2*), involved in glycerol assimilation and osmoprotection, was exclusively upregulated (LFC = 6.58). This gene helps cells retain water by metabolizing glycerol, thus combating salt-induced osmotic stress without impairing development (Martin-Hernandez et al., 2023; Carly et al., 2017). Additionally, a significant enhancement of mitochondrial energy production was observed, marked by strong upregulation of *Zm00001eb260760* (*AAE2*), an acyl-activating enzyme essential for cellular respiration (Hooks et al., 2012; Jin et al., 2021; Cheng et al., 2018), supporting germination under high energy demands.

MERCK also appeared to balance defense with developmental programs. The upregulation of *Zm00001eb411430*, a *beta-glucosidase* (*BGLU*) involved in activating stored phytohormones and chemical defense compounds (Gan et al., 2022; Gomez-Anduro et al., 2011), suggests an enhanced chemical defense, potentially through the release of active ABA to promote drought tolerance (Ren et al., 2019). Furthermore, *Zm00001eb074830* (*HAT1*/*HAT2*), a maize ortholog from the *HD-ZIP II* transcription factor family, was strongly upregulated. This family typically represses ABA-mediated drought responses while promoting auxin- and GA-mediated growth (Yuan et al., 2021; Kang et al., 2025), this expression pattern suggesting a pro-growth strategy that suppresses full-blown stress responses to protect hormonal balance necessary for successful germination and establishment.

#### 4.2.3 ABG treatment: maladaptive response and energy conservation

The ABG polymer treatment was associated with a transcriptomic profile suggestive of metabolic dysfunction, which may contribute to its intermediate performance. While stress-associated RNA silencing pathways (e.g., *Zm00001eb102530*, a *DRB5* homolog) were activated similarly to Merck, the regulatory control appeared to diverge, potentially resulting in an overwhelmed physiological state.

Metabolic dysfunction was suggested by the upregulation (LFC = 3.43) of *Zm00001eb337870* (*Sccinate Dehydrogenase*, *SDH*), a key enzyme linking the TCA cycle to the mitochondrial electron transport chain and a major source of mitochondrial ROS (mtROS) (Zhang et al., 2020; Jardim-Messeder et al., 2015). Its dysregulation has been shown to lead to excessive mtROS and impaired growth (Belt et al., 2017). In our study, this upregulation correlated with the lowest TPC, potentially indicating phenolic depletion. This inferred redox imbalance may contribute to ABG’s reduced physiological performance, as excessive mtROS may not have been sufficiently buffered by antioxidants.

Under ABG, *Zm00001eb019640*, a *PAT1*-subfamily *GRAS* regulator, is markedly downregulated (LFC= -3.42), indicating weaker activation of stress-response pathways and reduced expression of their downstream target genes. *GRAS*/*PAT1* factors are broadly stress responsive in cotton (Zhang et al., 2018), and *GRAS* members enhance salt tolerance in sweet orange (Ren et al., 2025). Consistently, ABG seedlings show poorer physiology and elevated Na^+^, together with reduced expression of ion-homeostasis/barrier modules (*SOS/NHX/HKT, suberin*) (Waseem et al., 2022; Neves et al., 2023). While causality in maize remains to be tested, lower *Zm00001eb019640* expression is compatible with impaired salt-stress signaling and ion balance under ABG.

In parallel, ABG-treated seedlings appeared to adopt a developmental shutdown survival strategy. *Zm00001eb360940* (*MAPKKK17*), a component of a *MAPK* cascade involved in developmental processes (Wang et al., 2015; Ye et al., 2017), was significantly downregulated. This repression of a key developmental gene suggests a strategic shutdown of non-essential growth programs, diverting resources toward survival. This putative adaptation aligns with ABG’s intermediate performance, where growth appeared to be limited in favor of survival (Wang et al., 2024).

#### 4.2.4 SWT treatment: catastrophic regulatory and developmental collapse

The severe phytotoxicity and marked developmental impairment observed with the SWT treatment, characterized by poor germination and high rates of abnormality, are consistently reflected in a transcriptomic signature indicative of systemic regulatory and developmental dysregulation. The lower sodium and TPC levels observed (**Figure 2**) may therefore be interpreted as symptoms of cellular dysfunction, rather than reduced stress. Similar to ABG, SWT treatment also downregulated transcription factors and defense pathways. Specifically, *Zm00001eb129690* (*WRKY47*), a transcription factor essential for combating various stresses in diverse plant species (Khoso et al., 2022; Singh et al., 2025), was significantly downregulated (LFC= -8.97). This downregulation suggests a disruption of the transcriptional control required for seedling establishment. This potential vulnerability was further compounded by the intense repression of *Zm00001eb093580* (ortholog of *DTX20*), a *MATE* transporter implicated in for ABA transport and protective responses (Lu et al., 2019; Jarzyniak et al., 2014). The observed low expression of these critical defense and hormone transport genes is consistent with the high vulnerability of SWT seedlings.

Furthermore, regulatory dysregulation appeared to extend to genes governing protein function. The maize zinc finger ortholog *Zm00001eb333860*, typically induced under abiotic stress and known for its role in stress tolerance (Yoon et al., 2014; Xu et al., 2008; Wang et al., 2008; Han et al., 2022), was strongly downregulated (LFC= -3.16). This expression pattern suggests a significant impairment indicating a profound failure in transcriptional defense. Additionally, *Zm00001eb095050*, a *RING/U-box* superfamily protein (E3 ubiquitin ligase) involved in the *ubiquitin-proteasome system* (*UPS)* for post-translational regulation (Adler et al., 2017; Yu et al., 2016; Wang et al., 2016, 2019; Liu et al., 2024), was also strongly repressed (LFC= -8.34). This repression suggests a reduced capacity to execute targeted protein degradation, which is necessary for reconfiguring cellular processes during stress. The combined downregulation of these key regulators offers a plausible molecular basis for the SWT phenotype. A compromised cellular network, lacking proper ion transport and protective compound synthesis, may have contributed to the observed developmental impairment and stress-induced damage.

### 4.2 Study limitations and outlook

While this study offers valuable initial insights into maize transcriptomic responses to SAPs under drought, it has limitations. Our investigation used a single maize cultivar and two biological replicates per treatment. Furthermore, only three SAP formulations were evaluated, limiting insights into the full diversity of polymer chemistries. Future research should address these by incorporating diverse maize genotypes and increasing replication for enhanced statistical power. Broader screening of additional SAP chemistries is also crucial. Ultimately, future work should integrate multi-year, multi-site field trials with mechanistic studies to: (i) verify agronomic benefits of promising SAP formulations, (ii) identify the precise source of Na^+^ release (which appears linked to abnormal seedling development), and (iii) functionally validate candidate gene biomarkers via techniques like qPCR or CRISPR/Cas9. This validation is key for developing high-throughput screening platforms. It will help optimize SAP technologies for broader applicability across crops and combined-stress environments, complementing genetic improvements for durable drought resilience.

## Data Availability

The raw data of gene expression levels (FeatureCount files) generated and analyzed for this study are available in Figshare, accessible via the link: https://doi.org/10.6084/m9.figshare.30165079.v1.
