# Supplementary figures and images for: Transcriptomic and physiological effects of superabsorbent polymer seed coating on maize under drought stress
Source: Front Plant Sci. 2026 Feb 5;17:1736004. doi: 10.3389/fpls.2026.1736004 (PMC12916425; doi:10.3389/fpls.2026.1736004)

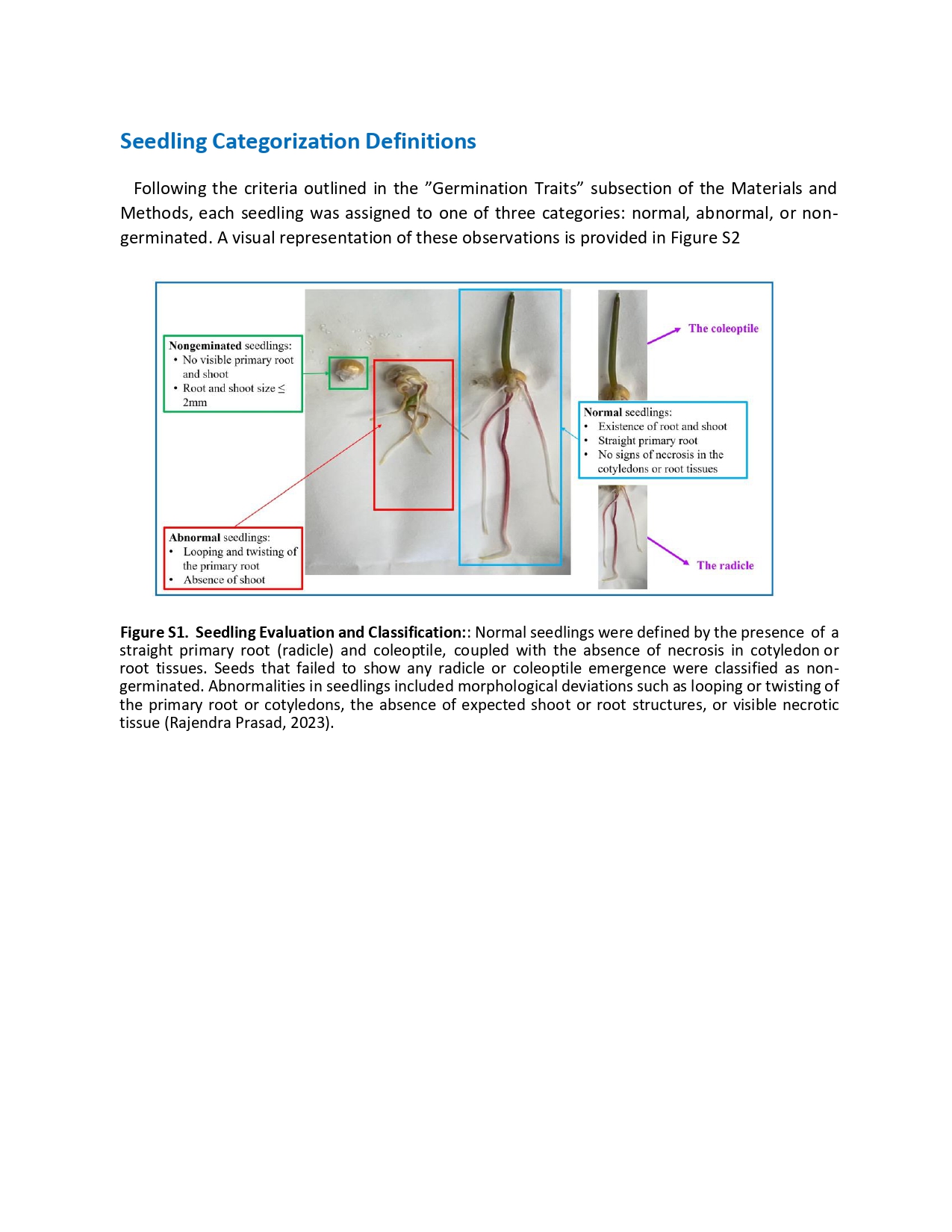

Supplement: Supplementary file 1 [file Image1.jpeg]

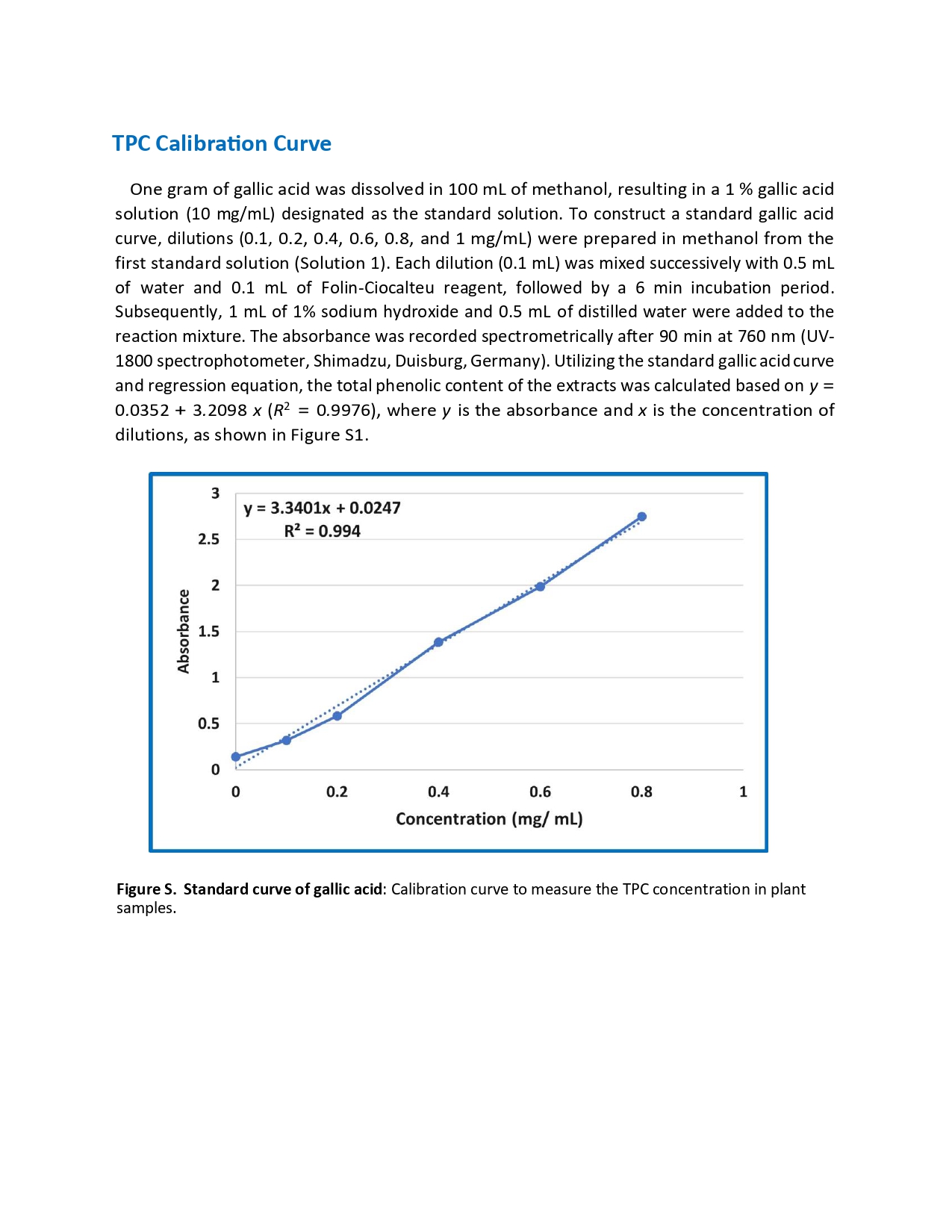

Supplement: Supplementary file 2 [file Image2.jpeg]
