# Supplementary material for: Transcriptomic and physiological effects of superabsorbent polymer seed coating on maize under drought stress
Source: Front Plant Sci. 2026 Feb 5;17:1736004. doi: 10.3389/fpls.2026.1736004 (PMC12916425; doi:10.3389/fpls.2026.1736004)
Supplement: Supplementary file 3 [file Table1.docx]

## **Comparison of SAP characteristics and application rates**

Table [S1](#_bookmark1) summarizes the key characteristics and application rates of three superabsorbent polymer (SAP) products, MERCK, SWT, and ABG, used in the study. The SAPs vary substantially in their water absorption capacities and recommended usage rates. MERCK exhibited the highest theoretical water absorption capacity, followed by ABG, with values in the 10–40 mL/g range. Similarly, when tested with distilled water, MERCK maintained a high capacity of 470 mL/g, while SWT dropped to 250 mL/g, and

ABG remained at only 10 mL/g. This highlights notable discrepancies between the theoretical and actual water absorption capacities of the tested products, with absorption performance in distilled water markedly lower than theoretical estimates, particularly for SWT.

These differences in absorption efficiency were also reflected in their application rates. For seed coating (based on 4 kg of seeds per hectare), ABG required the highest amount at 4,516 g, followed by 226 g for SWT and 113 g for MERCK. For soil application, ABG again required a substantially higher amount, whereas SWT and MERCK required lower but comparable amounts. These data indicate that although ABG has a much lower absorption efficiency, it is applied in significantly larger quantities, likely to compensate for its limited capacity to retain water compared to MERCK and SWT.

**Table S1.** **Comparison of water absorption capacity and application rates of different superabsorbent polymers (SAPs).** The table summarizes the theoretical and deionized (DI) water absorption capacities (mL/g) of three superabsorbent polymers (SAPs: MERCK, SWT, ABG) and their recommended per-hectare rates for seed coating and soil application, where soil application denotes incorporation of the SAP into the field soil. Rates are calculated for approximately 25 kg of seed.

| **SAP** | **Water absorption capacity (mL/g)** | | **Usage amount per hectare (25 kg seeds)** | |
| --- | --- | --- | --- | --- |
|  | **Theoretical** | **Observed** | **Seed coating (kg)** | **Soil application (kg)** |
| MERCK | 500 | 470 | 0.75 | 50-70 |
| SWT | 600 | 250 | 1.5 | 65 |
| ABG | 10-40 | 10 | 172.176 | 400 |
