# Supplementary material for: Transcriptomic and physiological effects of superabsorbent polymer seed coating on maize under drought stress
Source: Front Plant Sci. 2026 Feb 5;17:1736004. doi: 10.3389/fpls.2026.1736004 (PMC12916425; doi:10.3389/fpls.2026.1736004)
Supplement: Supplementary file 4 [file Table2.docx]

## Germination Rate Analysis

The table presents the proportions of normal, abnormal, and non-germinated seedlings for each treatment: CN (Control), CS, MERCK, ABG, and SWT, along with their corresponding 95% confidence intervals (CI). The proportions were calculated by dividing the number of seedlings in each category by the total number of seedlings for each treatment.

The Confidence Intervals (CI) were computed using the standard error (SE) for each proportion, which is given by:

*SE* = *p*(1 *− p*)

*n*

r

where *p* is the proportion of the category and *n* is the total number of seedlings. The 95% CI was calculated as:

*CI* = *p ±* 1*.*96 *× SE*

This CI provides a range within which the true proportion is expected to lie with 95% confidence.

The Risk Difference (RD) compares the proportion of seedlings in each category between the treatment and the control (CN). It was calculated as:

*RD* = *p*treatment *− p*CN

A positive RD indicates a higher proportion in the treatment compared to the control, while a negative RD indicates a lower proportion. For instance, the RD for **Normal** seedlings in **ABG** is -0.23, indicating a 23% lower proportion of normal seedlings in ABG compared to CN.

This analysis provides insights into how each treatment affects seedling germination compared to the control.

**Table S2. Proportions of Normal, Abnormal, and Non-germinated Seedlings Across Treatments with Confidence Intervals (CI) and Risk Differences (RD) Compared to Control (CN).** This table presents the proportions of normal, abnormal, and non-germinated seedlings across five treatments. For each treatment, the proportion of seedlings in each category is shown along with the 95% confidence intervals (CI). Additionally, the Risk Differences (RD) are reported for each treatment compared to the CN control treatment. Positive RD values indicate a higher proportion of seedlings in a given category for the treatment compared to CN, while negative RD values suggest a lower proportion.

| Treatment | Normal | | Abnormal | | Nongerminated | | RD: Reference treatment (CN) | | |
| --- | --- | --- | --- | --- | --- | --- | --- | --- | --- |
|  | Proportion | CI: Lower; Upper | Proportion | CI: Lower; Upper | Proportion | CI: Lower; Upper | Normal | Abnormal | Nongerminated |
| CN | 1.00 | 1.00; 1.00 | 0.00 | 0.00; 0.00 | 0.00 | 0.00; 0.00 | 0.00 | 0.00 | 0.00 |
| CS | 0.80 | 0.66; 0.94 | 0.17 | 0.03; 0.30 | 0.03 | -0.03; 0.10 | -0.20 | 0.17 | 0.03 |
| MERCK | 0.80 | 0.66; 0.94 | 0.13 | 0.01; 0.25 | 0.07 | -0.02; 0.16 | -0.20 | 0.13 | 0.07 |
| ABG | 0.77 | 0.62; 0.92 | 0.20 | 0.06; 0.34 | 0.03 | -0.03; 0.10 | -0.23 | 0.20 | 0.03 |
| SWT | 0.60 | 0.42; 0.78 | 0.30 | 0.14; 0.46 | 0.10 | -0.01; 0.21 | -0.40 | 0.30 | 0.10 |
