# Supplementary material for: Transcriptomic and physiological effects of superabsorbent polymer seed coating on maize under drought stress
Source: Front Plant Sci. 2026 Feb 5;17:1736004. doi: 10.3389/fpls.2026.1736004 (PMC12916425; doi:10.3389/fpls.2026.1736004)
Supplement: Supplementary file 5 [file Table3.docx]

## **Transcriptome analysis summary**

A total of 129 million to 177 million raw reads were obtained across five treatments, with two biological replicates per SAP treatment and control groups (Table [S3).](#_bookmark3) After adapter removal and filtering of low- quality sequences, over 97% of the resulting reads were classified as high-quality. When mapped to the *Zea mays* reference genome, between 92.16% and 95.04% of the high-quality sequences were successfully aligned, indicating efficient mapping to the reference genome.

**Table S3.** **mRNA-seq Read and Alignment Summary of Maize**. Summary of mRNA-seq read statistics for maize seedlings under different SAP treatments. Pre-filtering and post-filtering columns indicate total raw reads and reads retained after quality control, respectively. “Reads passed filters (%)” represents the proportion of reads retained after filtering, and “Alignment rate (%)” indicates the percentage of filtered reads successfully aligned to the reference genome.

| Sample | Treatment | Pre-filtering | Post-filtering | Reads passed filters (%) | Alignment rate (%) |
| --- | --- | --- | --- | --- | --- |
| MABG401 | ABG | 142,662,326 | 140,031,838 | 98.16 | 94.2 |
| MABG402 | ABG | 177,901,336 | 173,211,356 | 97.36 | 93.5 |
| MCN102 | CN | 159,794,982 | 156,511,131 | 97.94 | 94.7 |
| MCN103 | CN | 154,232,342 | 150,373,866 | 97.5 | 93.8 |
| MCS201 | CS | 144,096,598 | 141,006,498 | 97.86 | 94.1 |
| MCS202 | CS | 129,127,304 | 126,120,124 | 97.67 | 93.4 |
| MMERCK502 | MERCK | 169,713,132 | 165,234,846 | 97.36 | 93.9 |
| MMERCK503 | MERCK | 153,565,066 | 149,203,344 | 97.16 | 92.2 |
| MSWT301 | SWT | 134,458,074 | 131,798,134 | 98.02 | 93.9 |
| MSWT302 | SWT | 155,089,966 | 151,944,028 | 97.97 | 95 |
